# Dicalcium Silicate Induced Proinflammatory Responses through TLR2-Mediated NF-*κ*B and JNK Pathways in the Murine RAW 264.7 Macrophage Cell Line

**DOI:** 10.1155/2018/8167932

**Published:** 2018-05-02

**Authors:** Shixiang Lai, Liangjiao Chen, Wei Cao, Shiman Cui, Xingyang Li, Wenchao Zhong, Mingyu Ma, Qingbin Zhang

**Affiliations:** ^1^Key Laboratory of Oral Medicine, Guangzhou Institute of Oral Disease, Stomatology Hospital of Guangzhou Medical University, Guangzhou 510140, China; ^2^The Second Affiliated Hospital of Guangzhou Medical University, Guangzhou 510260, China

## Abstract

Proinflammatory responses are important aspects of the immune response to biomaterials, which may cause peri-implantitis and implant shedding. The purpose of this study was to test the cytotoxicity and proinflammatory effects of dicalcium silicate particles on RAW 264.7 macrophages and to investigate the proinflammatory response mechanism induced by C_2_S and tricalcium phosphate (TCP). C_2_S and TCP particles were characterized using scanning electron microscopy (SEM), energy spectrum analysis (EDS) and X-ray diffraction (XRD). Cytotoxicity and apoptosis assays with C_2_S and TCP in the murine RAW 264.7 cell line were tested using the cell counting kit-8 (CCK-8) assay and flow cytometry (FCM). The detection results showed that C_2_S and TCP particles had no obvious toxicity in RAW 264.7 cells and did not cause obvious apoptosis, although they both caused an oxidative stress response by producing ROS when the concentrations were at 100 *μ*g/mL. C_2_S particles are likely to induce a proinflammatory response by inducing high TLR2, TNF-*α* mRNA, TNF-*α* proinflammatory cytokine, p-I*κ*B, and p-JNK1 + JNK2 + JNK3 expression levels. When we added siRNA-TLR2-1, a significant reduction was observed. These findings support the theory that C_2_S particles induce proinflammatory responses through the TLR2-mediated NF-*κ*B and JNK pathways in the murine RAW 264.7 macrophage cell line.

## 1. Introduction

An important factor of biological material safety evaluation is to explore the toxicity effects of biological materials on cells and their proinflammatory effects. We previously reported that following coculture with the murine RAW 264.7 cell line, dicalcium silicate coating material and silicon, calcium, and other released ions showed no obvious cytotoxicity effects on RAW 264.7 cells; however, there was a potential proinflammatory response, and it increased the release of inflammatory factors [1].

Dicalcium silicate (Ca_2_SiO_4_) is an important material in the calcium-silica system, which is frequently identified as an important constituent in Portland cement [2, 3]. Previous studies have indicated that *γ*-Ca_2_SiO_4_ ceramic possesses good bioactivity, biocompatibility, and mechanical properties and that it might be a promising bone implant material [4, 5]. Additionally, *γ*-Ca_2_SiO_4_ ceramic might be suitable for a potential application in the biomedical field, preferentially as materials for bone repair [6]. Some studies demonstrated that Ca_2_SiO_4_ powder ceramics and coatings are bioactive and can quickly induce the formation of a bone-like apatite layer on their surface after soaking in simulated body fluid (SBF) [7, 8]. Dicalcium silicate (C_2_S) and tricalcium phosphates (TCP) can both develop desirable physical properties as a result of undergoing hydration reactions [9], both of which were studied in this report.

Tricalcium phosphate ((Ca_3_(PO_4_)_2_) = TCP) is one of the most important biomaterials based on phosphates, and once it is mixed with a liquid phase, it produces much more rapid bone growth and union, currently recognized as ceramic material, which significantly simulates the mineralogical structure of bone [10, 11] When TCP ceramics are implanted in vivo, they are nontoxic, antigenically inactive, and noncarcinogenic, and they bond directly to bone without intervening connective tissue. They show no cytotoxicity and good biological compatibility and osteoconductivity in living tissues, and they are therefore used clinically as a hard tissue replacement [12–14]. Ca_2_SiO_4_-Ca_3_(PO_4_)_2_ ceramics are cytocompatible, and they are able to induce the osteoblastic differentiation of undifferentiated adult mesenchymal stem cells of human origin(ahMSCs) [15]. The ionic products of plasma-sprayed dicalcium silicate coating are beneficial to the proliferation and differentiation of MG63 osteoblast-like cells, and they differentially regulated osteoclastogenic gene expression by upregulating OPG and downregulating RANKL [16, 17].

The cell attachment, cytotoxicity, antibacterial efficacy, bioactivity, and biocompatibility properties of dicalcium silicate cement consistently make it a potential candidate as a root-end filling material and root sealer for endodontic use [18–20].

However, as for the mechanism of the dicalcium silicate proinflammatory response, there is no relevant research. This study aimed to detect the proinflammatory factors released from dicalcium silicate and tricalcium phosphate after being cocultured with RAW 264.7 cells and to explore their proinflammatory response mechanisms. Deeply exploring the mechanism of dicalcium silicate proinflammatory response can help further provide an understanding of the potential risks and clinical applications and provide certain references for the extensive use of dicalcium silicate materials in the clinic.

## 2. Materials and Methods

### 2.1. Materials

*β*-Dicalcium silicate (*β*-Ca_2_SiO_4_) was synthesized in the Shanghai Institute of Ceramics laboratory at the Chinese Academy of Sciences [7]. *β*-Tricalcium phosphate (Sigma, USA) was purchased from Sigma.

### 2.2. The Characterization of Dicalcium Silicate and Tricalcium Phosphate

Surface morphologies were observed using scanning electron microscopy (SEM, Hitachi S-3400N, Japan), and elemental analyses were detected with an energy dispersive X-ray spectroscopy (EDS, IXRF system, Model 550i, USA). The crystal structure was analysed using X-ray diffraction (XRD, Empyrean XRD system, PANalytical B.V., Dutch), and the diffraction pattern was measured in the 2*θ* range from 10° to 80°. The size of the two particles types was detected using laser particle size analysis (MS2000, Melvin Instrument Co. Ltd, England).

### 2.3. Estimation of Endotoxin Levels on C_2_S and TCP Particles

Packaging procedures were subject to continuous endotoxin control. Particle endotoxin levels were measured using a limulus reagent, bacterial endotoxins, and the maximum valid dilution (Xiamen Limulus Reagent Laboratory Co. Ltd, Fujian, China) to exclude the possibility of proinflammatory effects arising from the bacterial contamination of the C_2_S and TCP particles. The particles were dropped into endotoxin-free water and incubated with *Limulus polyphemus* in sterilized glass tubes at 37°C, and the gel formation was evaluated after 1 h.

### 2.4. Cell Culture

The mouse macrophage cell line, RAW 264.7 (American Type Culture Collection, TIB71, MD, USA) was used to evaluate the cytotoxicity and cytokine production induced by the C_2_S and TCP. The cells were maintained in DMEM supplemented with 10% foetal calf serum (Life Technologies, USA), under a saturated 5% CO_2_ and 95% air atmosphere. The cells were cocultured with C_2_S and TCP. The negative controls without any material were also examined. The culture medium containing 0.64% phenol solution was used as a positive control to evaluate cell viability. The cells cultured in DMEM with 5 *μ*g/mL lipopolysaccharide (LPS, Sigma, St. Louis, USA) were used as a positive control to evaluate cytokine production.

### 2.5. Cytotoxicity and Apoptosis Profiles in Response to Dicalcium Silicate and Tricalcium Phosphate

Murine RAW 264.7 cells were inoculated into 96-well plates with well densities of 6000. Additionally, we set up a blank medium group, a medium + cells group, and 10 *μ*g/mL and 100 *μ*g/mL dicalcium silicate and tricalcium phosphate particle groups and incubated the cells for 6, 24, 48, and 72 hours. When measuring apoptosis, RAW 264.7 cells were inoculated into 6-well plates at a cell density of 1 × 10^6^. Additionally, we set up a blank cell group, and we incubated cells with 10 *μ*g/mL or 100 *μ*g/mL concentrations of two kinds of dicalcium silicate and tricalcium phosphate particles as well as a positive control group. All six groups were incubated for 24 hours. The positive control group has no material, but it included a medium containing a 0.64% phenol solution. The cell vitality was evaluated using the cell counting kit-8 assay (CCK 8, BestBio, China).

According to the FITC Annexin V apoptosis detection kit description (BD Pharmingen™), the dead RAW 264.7 cells stained with both FITC Annexin V and PI. When apoptosis is measured over time, the cells can be often tracked as FITC Annexin V and PI negative (viable or no measurable apoptosis), to FITC Annexin V positive and PI negative (early apoptosis, membrane integrity is present), and finally to FITC Annexin V and PI positive (end stage apoptosis and death). The movement of cells through these three stages suggests apoptosis. The instrument model we utilized was a BD FACS Canto™ II flow cytometer. After the samples were run, we used the BD FACSDiva software to dispose the result and obtain data result plots.

### 2.6. Oxidative Stress Response

To explore the oxidative stress response of the C_2_S and TCP particles in the RAW 264.7 cells, we used a reactive oxygen species (ROS) test kit (Nanjing Jiancheng Biology Engineering Institute, Jiangsu, China) to test the superoxide dismutase (SOD) activity. An inverted fluorescence microscope and flow cytometry (FCM) were used to evaluate the ROS amounts. RAW 264.7 cells were cultured in 6-well plates, and we set up groups that included a negative control group, 10 *μ*g/mL or 100 *μ*g/mL C_2_S and TCP particles groups, and a positive control group, in which we added H_2_O_2_. We extracted the cells and inspected them after they had been incubated for 6 hours.

### 2.7. siRNA Interference of Relative TLR2 mRNA Expression

We entrusted Guangzhou Ribobio Biology Technology Company, Ltd (Ribobio) to design and synthesize three small interfering RNAs (siRNA) to interfere with TLR2 mRNA expression. The primer sequences we used for the siRNA are listed in Table 1. According to the product manual operation, we selected and utilized a 50 nM siRNA concentration. Murine RAW 264.7 cells were inoculated into 6-well plates at a cell density of 8 × 10^5^ cells. The reaction system in each well included 120 *μ*L riboFECT™ CP Buffer, 5 *μ*L siRNA, and 12 *μ*L riboFECT CP Reagent. Then, we added three different siRNAs to three different wells to test the interference efficiency. Next, we used qRT-PCR to determine the TLR2 mRNA gene expression after 48 h. Here, we utilized five groups, including negative control, positive control, siRNA-TLR-1, siRNA-TLR-2, and siRNA-TLR-3 groups. The fluorescent siRNA was used to transfect the RAW 264.7 cells for 48 h. Then, we compared the fluorescent cell numbers through an inverted fluorescence microscope with total cell amounts with a light microscope, in which the transfection efficiency of the transfection reagent could be evaluated more intuitively.

### 2.8. Quantitative Real-Time Polymerase Chain Reaction (qRT-PCR) Analyses of Steady-State mRNA Levels of TLR2, TNF-*α*, IL-1*β*, and IL-6

RAW 264.7 cells were cultured in 6-well plates, and we set up groups that included a control group, a siRNA-control group, 10 *μ*g/mL or 100 *μ*g/mL C_2_S and TCP groups, 10 *μ*g/mL or 100 *μ*g/mL C_2_S and TCP groups plus siRNA-TLR2-1, and a 5 *μ*g/mL LPS group, which were cultured for 24 h. After 24 h, qRT-PCR was used to detect the relative TLR2, TNF-*α*, IL-1*β*, and IL-6 mRNA expression levels. The total RNA was extracted using RNAiso Plus (Takara, Japan). RNA (1 *μ*g) was obtained from each sample and was reverse-transcribed using oligo-dT as the first-strand cDNA primer (revert aid first strand cDNA synthesis kit, Thermo Scientific, USA). TLR2, TNF-*α*, IL-1*β*, IL-6, and GAPDH primer sequences and their fragment lengths are shown in Table 2. The reverse-transcribed cDNA was subjected to q-PCR (SYBY Premix Ex Taq, Takara, Japan) using the following cycling conditions: 95°C for 10 min (initial denaturation), 40 cycles of 95°C for 15 s and 60°C for 60 s, and then 60°C for 5 min for terminal extension. The temperature was elevated one degree each 20 s to obtain a melting curve. The *ΔΔ*Ct method of relative quantification was used to determine the fold change in expression. Delta Ct (*Δ*Ct) represents the difference between the target Ct value and the control Ct value for each sample: *Δ*Ct = Ct (target gene) − Ct (control gene). The expression was further normalized using the control (*ΔΔ*Ct = ΔCt − Ct(control group). The fold change in expression was then obtained as 2^−*ΔΔ*Ct^, and a graph was plotted for 2^−*ΔΔ*Ct^.

### 2.9. Cytokine Production Measurements Using ELISA

After 24 h of incubation, the supernatants were collected and centrifuged at 1000 g for 15 min. TNF-*α*, IL-1*β*, and IL-6 were measured using commercial ELISA kits (Neobioscience Technology Company). The ELISA kit sensitivities were 15 pg/mL for TNF-*α*, 8 pg/mL for IL-1*β*, and 8 pg/mL for IL-6.

### 2.10. Western Blot Analysis for Relative Protein Expression

RAW 264.7 cells were inoculated into 6-well plates at a cell density of 1 × 10^6^ cells. Then, we added nothing but RAW 264.7 cells as a negative control group, 10 *μ*g/mL LPS as positive group, 10 *μ*g/mL or 100 *μ*g/mL C_2_S and TCP, and 10 *μ*g/mL or 100 *μ*g/mL C_2_S and TCP plus siRNA-TLR2-1 as experimental groups. The cells were lysed by RIPA lysis buffer with cocktail protease and phosphatase inhibitors at 4°C for 30 minutes. The cytoplasmic and nuclear proteins were obtained with a nuclear and cytoplasmic protein extraction kit (Beyotime) according to the manufacturer's instruction. The protein concentrations were determined with an enhanced BCA protein assay kit (Beyotime). Sixty micrograms of protein was separated via 10% SDS-PAGE and then transferred to positively charged nylon membranes. After transferring, the membranes were blocked with 5% bovine serum albumin (BSA) in TBS with 0.1% Tween-20 for 1 h at 37°C and then blotted at 4°C overnight with GAPDH, MyD88, p-p38, p-I*κ*B, or p-JNK1 + JNK2 + JNK3 primary antibodies. Specific bands were detected with ECL (Advansta, USA). The Quantity One software was used to quantify the band densities.

### 2.11. Statistical Analysis

Bar graphs were used to show the mean and standard deviation (SD) of triplicate experiments. The data were normalized, and data that passed the normality test were analysed using one-way ANOVA with the least significant difference (LSD) test. The Dunnett's test was utilized for abnormal distributions. All analyses were conducted using the SPSS 18.0 software (SPSS Inc., Chicago, IL, USA). *P* values < 0.05 were considered statistically significant.

## 3. Results

### 3.1. Characterization of Dicalcium Silicate and Tricalcium Phosphate Particle

**Figure 1:**
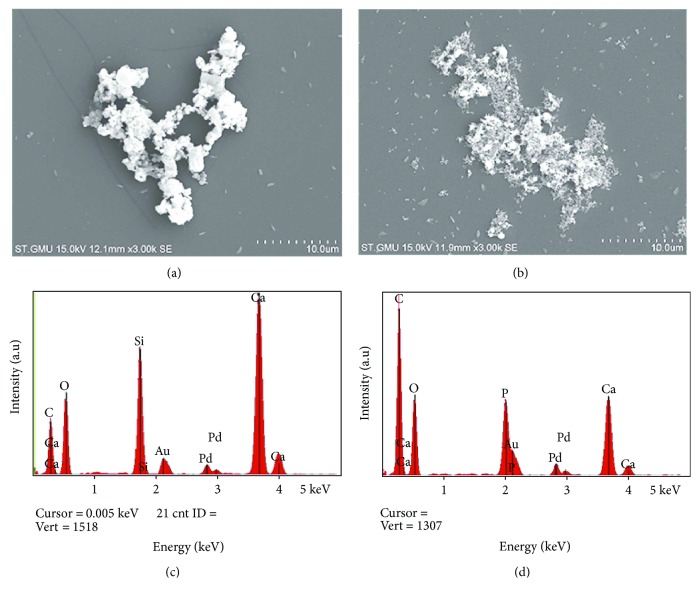
SEM photographs and EDS analysis of the two biomaterials. SEM shows the surface topography of the C_2_S and TCP particles (3000x), whereas the EDS analysis shows the elemental composition of the C_2_S and TCP particles. SEM photographs of (a) C_2_S and (b) TCP. EDS analyses of (c) C_2_S and (d) TCP.

SEM and EDS were used to evaluate the surface topography of C_2_S and TCP particles. Observed under SEM (3000x), the C_2_S particles presented as circular particles, but most of the particles aggregated together as a block mass, while the TCP hydrate under SEM had two different shapes: strip and grainy shapes (Figures 1(a) and 1(b)).

The EDS analysis showed that oxygen, carbon, calcium, and silica existed in the surface of dicalcium silicate particles, while carbon, calcium, phosphorus, and oxygen existed on the surface of the tricalcium phosphate particles (Figures 1(c) and 1(d)).

**Figure 2:**
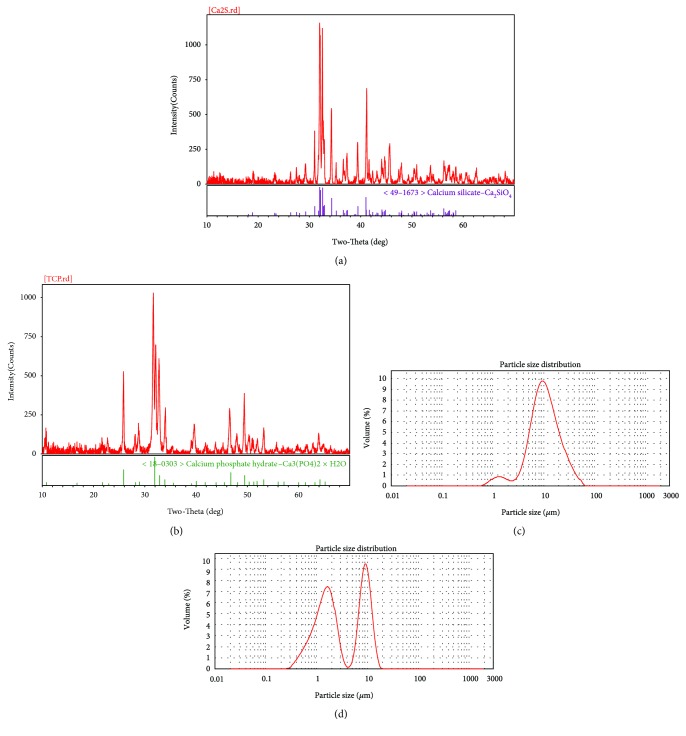
The XRD patterns of the two biomaterials and the size of the two particle types detected by particle size analysis: (a) XRD patterns of C_2_S, (b) XRD patterns of TCP, (c) the size distribution of the C_2_S particles, and (d) the size distribution of the TCP particles.

The XRD analysis showed that the dicalcium silicate particle collection of illustration matched <49–1673 > calcium silicate-matching Ca_2_SiO_4_ and the tricalcium phosphate particle collection of illustration matched <18–0303 > calcium phosphate hydrate − Ca_3_(PO_4_)_2_·H_2_O (Figures 2(a) and 2(b)).

The laser particle size analysis results showed that the C_2_S particles had one main size, approximately 8.710–10.000 *μ*m, while the TCP particles had two main sizes, approximately 1.660–1.905 *μ*m and 8.710–10.000 *μ*m (Figures 2(c) and 2(d)).

### 3.2. Estimation of Endotoxin Levels on C_2_S and TCP Particles

The particles were dropped into endotoxin-free water and incubated with *Limulus polyphemus* in sterilized glass tubes at 37°C, and the gel formation was evaluated after 1 h. No gel was generated in the control or in the 10 *μ*g/mL and 100 *μ*g/mL C2S and TCP particle groups, while the gel was produced in the positive control group, which contained bacterial endotoxin. The sensitivity of the *Limulus polyphemus* was 0.03 EU/mL.

### 3.3. Cytotoxicity and Apoptosis Profiles in Response to Dicalcium Silicate and Tricalcium Phosphate

**Figure 3:**
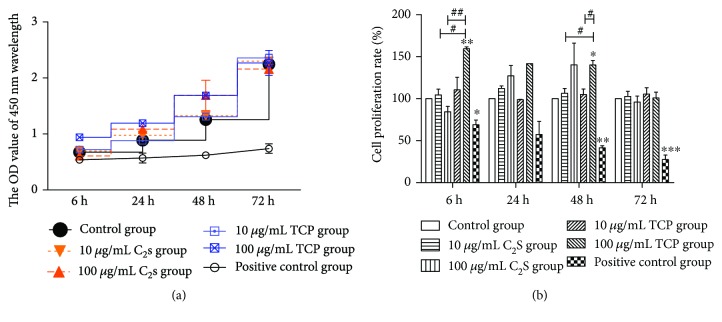
The OD values at 450 nm wavelength (a) and the cell proliferation rate (b) of the RAW 264.7 cells after being cocultured with C_2_S and TCP for 6 h, 24 h, 48 h, and 72 h. (a) The OD values at 450 nm wavelength of experimental groups were almost higher than in the control group. The positive control group showed low OD values at 450 nm wavelength. (b) More than 90% of the cells were viable in the experimental groups, and the proliferation rate was low in the positive control group. A higher proliferation rate was observed at 100 *μ*g/mL TCP compared with 10 *μ*g/mL (*P* < 0.05) and 100 *μ*g/mL C_2_S (*P* < 0.01) after being cocultured for 6 h. Additionally, 100 *μ*g/mL TCP produced a higher proliferation rate than the 10 *μ*g/mL of TCP (*P* < 0.05) and C_2_S (*P* < 0.05) cocultured for 48 h. ^∗^*P* < 0.05, ^∗∗^*P* < 0.01, and ^∗∗∗^*P* < 0.001 (experimental group versus control group). ^#^*P* < 0.05, ^##^*P* < 0.01, and ^###^*P* < 0.001 (C_2_S versus TCP).

To evaluate the cell proliferation and cell toxicity, we used a CCK-8 cell proliferation test kit (Betboy, China) in accordance with the manufacturer's instructions. RAW 264.7 cells were cultured with 10 *μ*g/mL and 100 *μ*g/mL C_2_S and TCP materials for 6 h, 24 h, 48 h, and 72 h. Following culture with these two particle types, the RAW 264.7 cell growth over time was not affected, no matter the particle concentration (10 *μ*g/mL or 100 *μ*g/mL), which indicated no obvious cytotoxicity (Figure 3). Following the CCK-8 proliferation test evaluation, over 90% of the cells were activated in the material groups (Figure 3).

**Figure 4:**
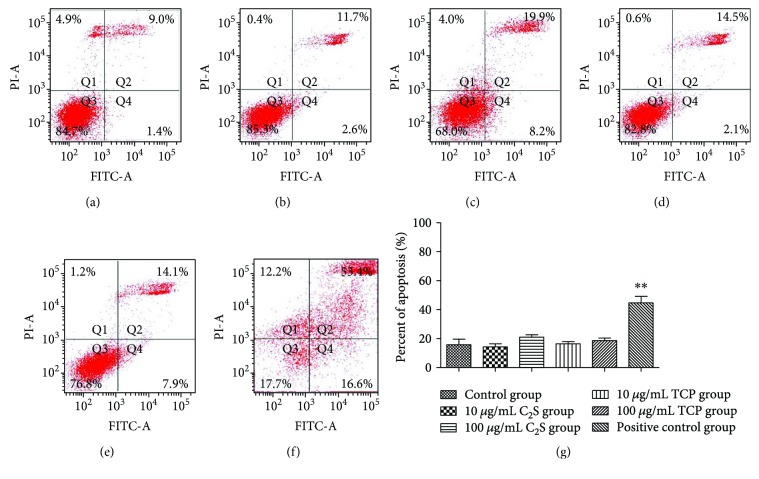
RAW 264.7 cells alone (a) and RAW 264.7 cells cultured with 10 *μ*g/mL C_2_S, 100 *μ*g/mL C_2_S, 10 *μ*g/mL TCP, 100 *μ*g/mL TCP, or 0.64% phenol solution (b, c, d, e, and f). Approximately 14.7% of the RAW 264.7 cells underwent apoptosis in the control group; approximately 14.3% and 20.9% of the cells underwent apoptosis in the 10 *μ*g/mL and 100 *μ*g/mL C_2_S particle groups, respectively; approximately 16.4% and 18.6% of the cells underwent apoptosis in the 10 *μ*g/mL and 100 *μ*g/mL of TCP groups, respectively. When the material concentrations were increased tenfold, no obvious apoptosis changes were observed. No significant difference was found amongst these groups (g) except for in the positive control group (44.7% cells underwent apoptosis compared with the control group, *P* < 0.01). All values are represented as the mean ± SD of triplicate experiments.

According to the description of the FITC Annexin V apoptosis detection kit (BD Pharmingen), the movement of cells through the three stages suggested apoptosis; therefore, we counted the FITC Annexin V positive and PI negative cells and the FITC Annexin V and PI positive cells.  Figure 4(g) shows that the apoptosis in the groups and whether there was a significant difference between them. Compared with the control group, there was no obvious difference when the cells were cocultured with 10 *μ*g/mL or 100 *μ*g/mL TCP or C_2_S; however, remarkable differences were observed between the negative and positive control groups (*P* < 0.01).

### 3.4. Oxidative Stress Response

According to the description of the ROS test kit, we used an inverted fluorescence microscope and FITC of FCM to evaluate the amount of ROS produced by the negative control, positive control, and 10 *μ*g/mL or 100 *μ*g/mL C_2_S or TCP particle groups.

**Figure 5:**
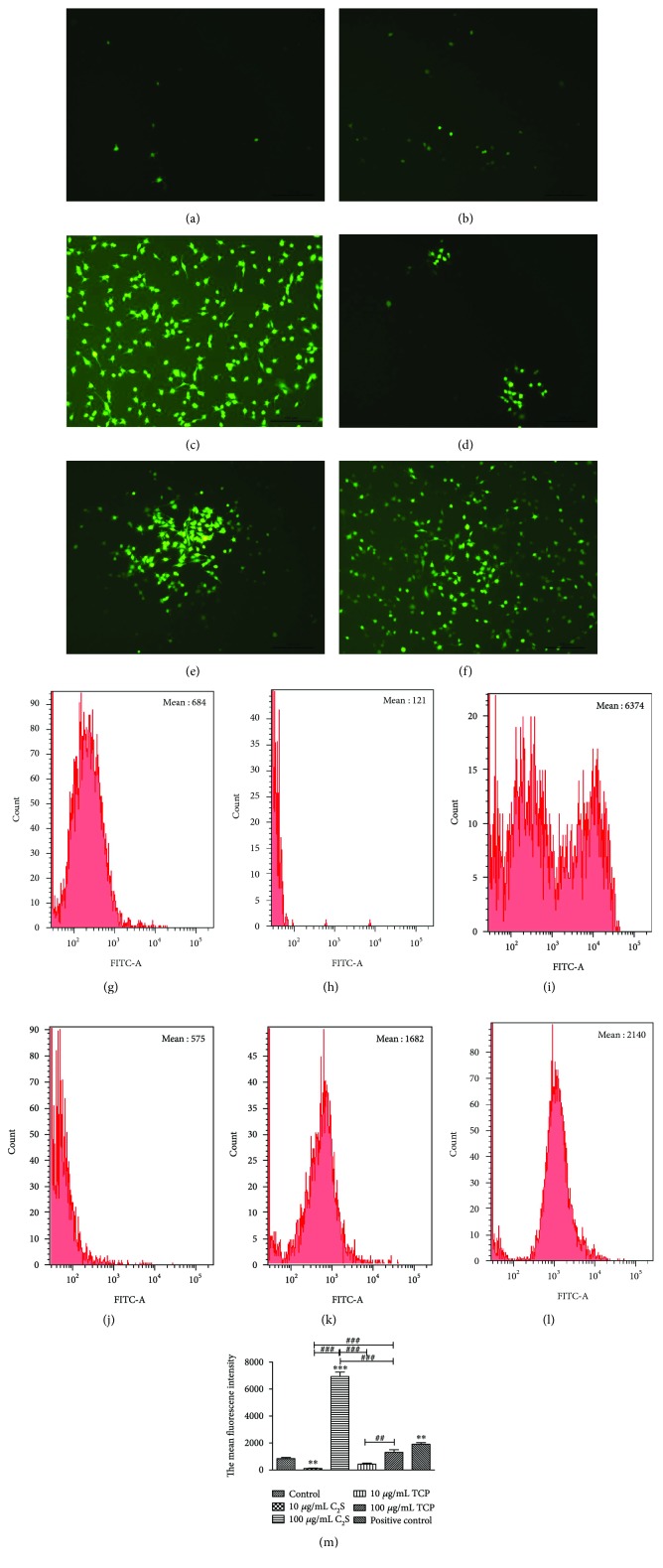
(a), (b), (c), (d), (e), and (f) were captured using an inverted fluorescence microscope, while (g), (h), (i), (j), (k), and (l) were acquired using FCM, which also produced the mean fluorescence intensity of the groups. (a and g) Control group, (b and h) 10 *μ*g/mL C_2_S particle group, (c and i) 100 *μ*g/mL C_2_S particle group, (d and j) 10 *μ*g/mL TCP particle group, (e and k) 100 *μ*g/mL TCP particle group, and (f and l) positive control group. (m) A cartogram of the mean fluorescence intensity. The 10 *μ*g/mL C_2_S (compared with control group, *P* < 0.01) and TCP particle groups showed no obvious ROS and had even lower levels than in the control group. However, when the concentration was brought up to 100 *μ*g/mL, a large amount of ROS was produced. Moreover, the 100 *μ*g/mL C_2_S particle group generated more ROS than the 10 *μ*g/mL (*P* < 0.001) and 100 *μ*g/mL (*P* < 0.001) TCP particle groups as well as the 10 *μ*g/mL C_2_S particle group, as shown above. Significant changes were observed between the 10 *μ*g/mL and the 100 *μ*g/mL TCP particle groups (*P* < 0.01) and the 10 *μ*g/mL C_2_S particle group (*P* < 0.001). The RAW 264.7 cells produced obvious ROS when incubated with H_2_O_2_ (*P* < 0.01). ^∗^*P* < 0.05, ^∗∗^*P* < 0.01, and ^∗∗∗^*P* < 0.001 (experimental group versus control group). ^#^*P* < 0.05, ^##^*P* < 0.01, and ^###^*P* < 0.001 (between the experimental groups).

As the result shows, compared with control group, which had a mean fluorescence intensity of 684, the 10 *μ*g/mL C_2_S and TCP particle groups showed hypofluorescence and produced less ROS, with mean fluorescence intensities of 121 and 575, respectively. While the 100 *μ*g/mL C_2_S and TCP particle groups showed hyperfluorescence and more obvious ROS, with mean fluorescence intensities of 6374 and 1682, respectively. In the positive control group, we observed hyperfluorescence and distinct ROS with a mean fluorescence intensity of 2140 (Figure 5).

### 3.5. siRNA Interfered with the TLR2 mRNA Expression

We use three siRNAs provided by the Ribobio Biology Company to interfere with the TLR2 mRNA expression. We directly observed the fluorescent and common cell amounts with an inverted fluorescence microscope to evaluate the transfection efficiency of the transfection reagents and detected the TLR2 mRNA expression levels to determinate the interference effect.

**Figure 6:**
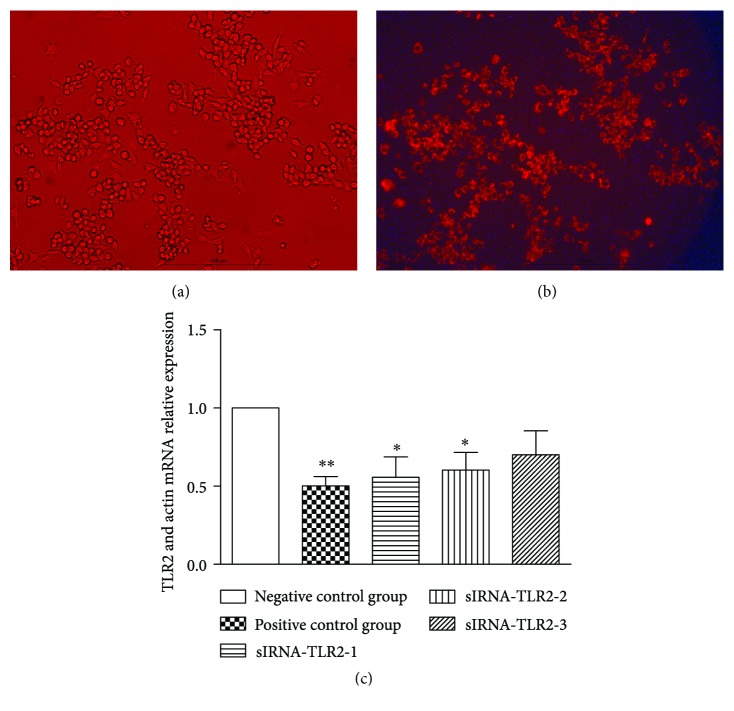
(a) RAW 264.7 cells with RNA oligo captured by a red light microscope. (b) RAW 264.7 cells with RNA oligo captured by an inverted fluorescence microscope. (c) The TLR2 mRNA expression in RAW 264.7 cells after siRNA-TLR2-2 interference. The positive group showed a significant decrease in TLR2 mRNA expression (*P* < 0.01). Additionally, both siRNA-TLR2-1 and siRNA-TLR2-2 had obvious interference effects (*P* < 0.05). ^∗^*P* < 0.05 and ^∗∗^*P* < 0.01. No special explanation means experimental versus control group.

Obviously, the number of fluorescent cells observed under the inverted fluorescence microscope (Figure 6(b)) was almost the same as the number of common cells observed under the ordinary red light microscope (Figure 6(a)). The TLR2 mRNA levels of positive control group were significantly decreased (*P* < 0.01). Both siRNA-TLR2-1 and siRNA-TLR2-2 had obvious interference effects, with efficiencies of approximately 56% and 60%, respectively, compared with the negative control groups. These differences were statistically significant (*P* < 0.05). The siRNA-TLR2-3 interference effect was not ideal compared with the negative control group. This difference was not statistically significant (Figure 6(c)). Therefore, we selected siRNA-TLR2–1, whose interference effect was the most ideal, in the follow-up studies.

### 3.6. The Relative Gene Expression

To determine the effects of the C_2_S particles on proinflammatory mediator expression in macrophages, TLR2, TNF-*α*, IL-1*β*, and IL-6 mRNA levels were tested using RT-qPCR. The cells were harvested 24 h after cultivation. The LPS-treated cells were used as positive controls.

**Figure 7:**
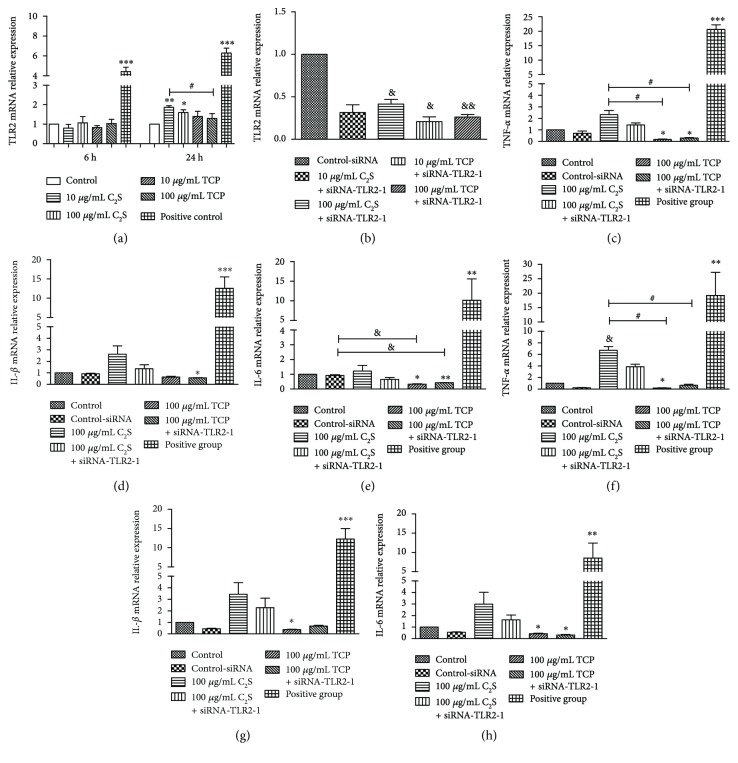
Relative mRNA gene expression. (a) and (b) represent TLR2 expression after being cocultured with 10 *μ*g/mL and 100 *μ*g/mL C_2_S or TCP for 6 h and 24 h in RAW 264.7 cells and the relative TLR2 expression after adding siRNA-TLR2-1. After 24 h, the C_2_S treatment increased the TLR2 expression more than the TCP treatment compared with the control group. Additionally, the TLR2 mRNA expression in the 10 *μ*g/mL C_2_S group was significantly higher than the 100 *μ*g/mL TCP group (*P* < 0.05). When we added siRNA-TLR2-1 to these groups, the TLR2 mRNA expression obviously decreased in the 100 *μ*g/mL C_2_S (*P* < 0.05), 10 *μ*g/mL TCP (*P* < 0.05), and 100 *μ*g/mL TCP groups (*P* < 0.01). (c), (d), (e), (f), (g), and (h) represent the TNF-*α*, IL-1*β*, and IL-6 expressions following coculture with 10 *μ*g/mL and 100 *μ*g/mL of C_2_S and TCP, respectively, in RAW 264.7 cells after 24 h. The 10 *μ*g/mL C_2_S treatment increased the TNF-*α* expression to a greater extent than TCP (*P* < 0.05). After the siRNA-TLR2-1 was added, the expression decreased (e). The 100 *μ*g/mL C_2_S produced high TNF-*α* mRNA levels (*P* < 0.05), which were higher than the 100 *μ*g/mL TCP treatment (*P* < 0.05) (h). The TNF-*α*, IL-1*β*, and IL-6 mRNA expression following the 10 *μ*g/mL and 100 *μ*g/mL TCP treatments were even lower than the control group (c, e, f, g, and h). The positive control group produced an obvious increase in TNF-*α*, IL-1*β*, and IL-6 mRNA expressions. The data are expressed as the mean ± SD. ^∗^*P* < 0.05, ^∗∗^*P* < 0.01, and ^∗∗∗^*P* < 0.001 (experimental group versus control). ^&^*P* < 0.05, ^&&^*P* < 0.01, and ^&&&^*P* < 0.001 (experimental group versus control-siRNA). ^#^*P* < 0.05, ^##^*P* < 0.01, and ^###^*P* < 0.001 (between the experimental groups).

The TLR2 mRNA levels in the RAW 264.7 cells were not obviously changed after being cocultured with 10 *μ*g/mL and 100 *μ*g/mL TCP and C_2_S for 6 h. A significant increase in TLR2 mRNA levels were observed for the 10 *μ*g/mL (*P* < 0.01) and 100 *μ*g/mL (*P* < 0.05) C_2_S particle groups after 24 h. The TLR2 mRNA expression in the 10 *μ*g/mL C_2_S group was significantly higher than the 100 *μ*g/mL TCP group (*P* < 0.05). A positive significant increase was observed (*P* < 0.001) (Figure 6(a)). When we added siRNA-TLR2-1 to the experimental groups, the TLR2 mRNA expression in the 100 *μ*g/mL C_2_S (*P* < 0.05), 10 *μ*g/mL TCP (*P* < 0.05), and 100 *μ*g/mL TCP (*P* < 0.01) groups obviously decreased (Figure 7(b)).

The LPS induced high levels of TNF-*α* expression after 24 h. After 24 h, the 100 *μ*g/mL C_2_S particle group significantly promoted TNF-*α* mRNA expression (*P* < 0.05). The TNF-*α* mRNA levels between the 10 *μ*g/mL and 100 *μ*g/mL C_2_S groups and the 10 *μ*g/mL and 100 *μ*g/mL TCP groups were significantly different (*P* < 0.05). Additionally, when we added siRNA-TLR2-1 to them, the TNF-*α* mRNA expression in the 10 *μ*g/mL and 100 *μ*g/mL C_2_S went down; however, no significant differences were observed between the 10 *μ*g/mL and 100 *μ*g/mL C_2_S + siRNA-TLR2-1 groups, the 10 *μ*g/mL and 100 *μ*g/mL TCP groups, or the 10 *μ*g/mL and 100 *μ*g/mL TCP + siRNA-TLR2-1 groups. The 10 *μ*g/mL and 100 *μ*g/mL TCP treatments produced low levels of TNF-*α* mRNA expression (*P* < 0.05) (Figures 7(c) and 7(f)).

Next, we examined IL-1*β* mRNA levels. No significant increase in IL-1*β* mRNA was observed in the C_2_S and TCP groups, no matter their concentration (i.e., 10 *μ*g/mL or 100 *μ*g/mL). The positive control group expressed high IL-1*β* mRNA levels after 24 h of cultivation (*P* < 0.001). The 100 *μ*g/mL TCP treatment produced low levels of IL-1*β* mRNA expression (*P* < 0.05) (Figures 7(d) and 7(g)).

Subsequently, IL-6 mRNA expression was detected (Figures 7(e) and 7(h)). Low levels of IL-6 mRNA were observed in the 10 *μ*g/mL and 100 *μ*g/mL TCP and C_2_S particle groups after 24 h (*P* < 0.05). At this time, high IL-6 mRNA levels were observed in the positive control group (*P* < 0.05). When we added siRNA-TLR2–1, the IL-6 levels slightly declined. No significant difference in IL-6 mRNA expression was observed between the C_2_S and TCP particle groups (*P* > 0.05). The 10 *μ*g/mL and 100 *μ*g/mL TCP treatments produced low IL-6 mRNA expression levels (*P* < 0.05) (Figures 7(e) and 7(h)).

### 3.7. Proinflammatory Cytokine Production Measured with ELISAs

**Figure 8:**
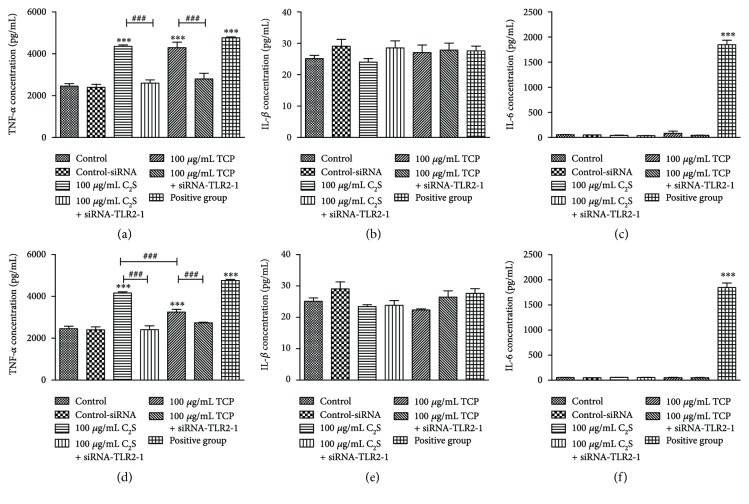
TNF-*α*, IL-1*β*, and IL-6 cytokine concentrations. (a, b, and c) TNF-*α*, IL-1*β*, and IL-6 concentrations of the control group, control-siRNA group, 10 *μ*g/mL C_2_S and TCP particle groups, 10 *μ*g/mL C_2_S + siRNA-TLR2-1 and TCP + siRNA-TLR2-1 groups, and positive control group. (d, e, and f) TNF-*α*, IL-1*β*, and IL-6 concentrations in the groups mentioned above; however, the material concentration was increased to 100 *μ*g/mL. Following incubation for 24 h, the 10 *μ*g/mL and 100 *μ*g/mL C_2_S and TCP particles produced high TNF-*α* levels (*P* < 0.001); however, when we added siRNA, the levels significantly decreased compared with the non-siRNA treated groups (*P* < 0.001). Moreover, the 100 *μ*g/mL C_2_S particle group produced more TNF-*α* than the 100 *μ*g/mL TCP particle group. There were no significant differences in IL-1*β* and IL-6 concentrations between the experimental groups. The positive control group produced high TNF-*α* and IL-6 levels (*P* < 0.001).

Based on our RT-qPCR results, ELISAs were used to detect the effects of C_2_S and TCP particles on TNF-*α*, IL-1*β*, and IL-6 proinflammatory cytokine production after 24 h. TNF-*α* concentrations were increased in the 10 *μ*g/mL and 100 *μ*g/mL C_2_S and TCP particle groups compared with the control group (*P* < 0.001). After we added siRNA-TLR2-1, the concentration decreased significantly (*P* < 0.001). No significant differences in TNF-*α* concentration were observed between the 10 *μ*g/mL C_2_S and TCP particle groups. Nevertheless, the 100 *μ*g/mL C_2_S particles induced a larger increase in TNF-*α* cytokine levels than the 100 *μ*g/mL TCP particles (*P* < 0.001) (Figures 8(a) and 8(d)).

Next, we examined IL-1*β* levels. There was no significant difference between the groups regarding the IL-1*β* concentration, including the group that added LPS (Figures 8(b) and 8(e)).

Finally, we measured the IL-6 concentration in all groups. The 10 *μ*g/mL and 100 *μ*g/mL C_2_S and TCP particle groups did not induce high IL-6 concentration levels, while the LPS strongly induced IL-6 expression (Figures 8(c) and 8(f)).

### 3.8. Relative Protein Expression

To explore the mechanism of proinflammatory mediator expression in RAW 264.7 cells, the relative GAPDH, myeloid differentiation factor88 (MyD88), p-p38, p-I*κ*B, and p-JNK1 + JNK2 + JNK3 protein expression levels were detected using Western blot analysis.

TLR signaling pathways can be largely classified as MyD88-dependent pathways, which recruits MyD88 paired with MyD88-like protein (Mal, also called TIRAP) and induces the production of inflammatory cytokines.

**Figure 9:**
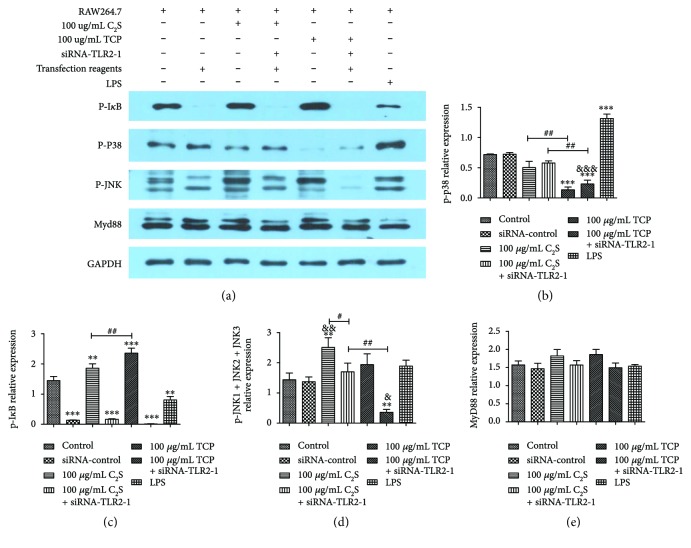
Relative protein expression. (a) represents the specific grouping situation and Western blot result of GAPDH, MyD88, p-p38, p-I*κ*B, and p-JNK1 + JNK2 + JNK3. (b), (c), (d), and (e) represent the relative expression of p-p38, p-I*κ*B, and p-JNK1 + JNK2 + JNK3 according to analysis of gray level differences. Compared with the control group, the p-p38 expression levels in the 100 *μ*g/mL TCP particle group and siRNA-TLR2-1 added group were significant lower (*P* < 0.001), and they showed, respectively, lower levels than the 100 *μ*g/mL C_2_S particle group with and without siRNA-TLR2-1. The positive control group showed high p-p38 expression levels (b). There was a significant difference in the p-I*κ*B levels between the control and 100 *μ*g/mL C_2_S particle groups (*P* < 0.01) as well as the TCP particle group (*P* < 0.001). Moreover, an obvious difference was observed between the 100 *μ*g/mL C_2_S and TCP particle groups (*P* < 0.01). The LPS induced low p-I*κ*B expression levels (*P* < 0.01) (c). The 100 *μ*g/mL C_2_S particle group expressed high p-JNK1 + JNK2 + JNK3 expression levels (*P* < 0.01), and they were obviously higher than when siRNA-TLR2-1 was added (*P* < 0.05). However, the 100 *μ*g/mL TCP + siRNA-TLR2-1 group showed low levels of p-JNK1 + JNK2 + JNK3 expression (*P* < 0.01) (d). No significant difference in MyD88 levels was observed in the groups, including in the LPS-treated group (e). The data are expressed as the mean ± SD. ^∗^*P* < 0.05, ^∗∗^*P* < 0.01, and ^∗∗∗^*P* < 0.001 (experimental group versus control). ^#^*P* < 0.05, ^##^*P* < 0.01, and ^###^*P* < 0.001 (between experimental groups).

First, we set up seven groups to test the relative GAPDH expression and made sure that the total protein levels in each group were almost the same (Figure 9(a)).

Then, the relative expression of the phosphorylated proteins, p-p38, p-I*κ*B, and p-JNK1 + JNK2 + JNK3 was tested to explore the proinflammation response mechanism. Compared with the control group, the 100 *μ*g/mL C_2_S particle group produced high p-I*κ*B (*P* < 0.01) and p-JNK1 + JNK2 + JNK3 expression levels (*P* < 0.01). When we added siRNA-TLR2-1, the expression significantly decreased. The 100 *μ*g/mL TCP particle group induced low p-p38 levels (*P* < 0.001) and high p-I*κ*B levels (*P* < 0.001); moreover, it induced lower p-p38 (*P* < 0.01) and higher p-I*κ*B levels (*P* < 0.01) than the 100 *μ*g/mL C_2_S particle group. When we added siRNA-TLR2-1, the 100 *μ*g/mL TCP particle group induced lower p-p38 (*P* < 0.01) and higher p-I*κ*B (*P* < 0.01) than the 100 *μ*g/mL C_2_S particle group. The LPS-treated group induced high p-p38 levels (*P* < 0.001) and low p-I*κ*B levels (*P* < 0.01) (Figures 9(b)–9(d)).

Lastly, the relative MyD88 expression levels were tested. No significant change in relative MyD88 expression was observed in the experimental and positive control groups. When we added siRNA-TLR2-1, no obvious decrease was observed (Figure 9(e)).

## 4. Discussion

Many research studies have been conducted on C_2_S, which have revealed that C_2_S materials exhibit many ideal characteristics. C_2_S and TCP have ideal physical properties by hydration reaction [9], and compared with TCP and hydroxyapatite (HAp), *β*-C_2_S has a higher strength value [21]. C_2_S materials have good biological activity and electrical conductivity [22, 23], and C_2_S is usually regarded as a potential coating material in orthopaedic and dental implant coating materials, because of its good biocompatibility and biological activity [24]. The study found that a new type of *α*′L + *β*-C_2_S_ss_ cement paste can support cell adhesion and diffusion and also found that it was beneficial to the early stage of bone regeneration. [25] Additionally, studies have found that calcium silicate ion release materials provide a good microenvironment for the survival and differentiation of human oral and maxillofacial mesenchymal stem cells (OFMSCs), and the combination of calcium silicate materials and OFMSCs can promote tissue regeneration for periapical bone defects [26].

Moreover, the potential applications of *α*′H-C_2_S dicalcium silicate material in medicine may be preferred as bone or dental restorative materials by studying the characteristics, biological activity, and biocompatibility of *α*′H-C_2_S dicalcium silicate bone cement doping with TCP [27]. Bone or dental restorative materials should not produce obvious inflammation reactions. However, most studies focused on the safety and osteogenesis effect of C_2_S ion products [28] and the interaction between material and immune cells. However, its potential proinflammatory response has not been explored, which limits its wide clinical applications.

TCP has not been extensively researched. Some scholars studied *β*-tricalcium phosphate porous bioceramics and found that after periodontal ligament cells (PDLCs) were cultured in vitro with *β*-TCP composite, they could proliferate and differentiate in the body and form a connective tissue, thus proving that it is feasible for *β*-TCP to act as scaffold materials in periodontal tissue engineering [29]. Some established a femoral head necrosis model and proved that it can be used in necrotic bone defect repair if compounded with bone marrow mesenchymal stem cells [30]. TCP coating can also delay the in vivo degradation of magnesium alloy, resulting in good biological activity [31]. The study of TCP has been relatively mature.

First, the materials were characterized using SEM, EDS, and XRD. The C_2_S particles we studied, a kind of micron material, presented as circular particles but most aggregated together as a block mass, and they consisted of oxygen, carbon, calcium, and silica elements. The TCP particles we studied, a kind of micron material, were observed as strip and grainy shapes in the SEM, and they were made up of carbon, calcium, phosphorus, and oxygen elements. The endotoxin levels on C_2_S and TCP particles were kept under 0.03 EU/mL. Next, the cytotoxicity and apoptosis profiles of the RAW 264.7 cells in response to the C_2_S and TCP particles were tested using CCK-8 kit and FITC Annexin V apoptosis detection kits. Most of the cells were viable after being cocultured with 10 *μ*g/mL and 100 *μ*g/mL C_2_S and TCP particles for 6 h, 24 h, 48 h, and 72 h. The 10 *μ*g/mL and 100 *μ*g/mL C_2_S and TCP particle treatments did not obviously lead to RAW264.7 cell apoptosis after 24 h.

Then, a ROS test kit was used to test the oxidative stress response of C_2_S and TCP particles in RAW 264.7 cells. ROS play a key role in cell metabolism and survival, as well as in the cytotoxicity mechanisms of various classes of carbon-based nanomaterials [32, 33]. To verify if the C_2_S and TCP particles induce an oxidative stress phenomena, DCFH–DA assays were performed [34, 35]. The titanium released from dental implant enhances the preosteoblast adhesion by ROS modulating crucial intracellular pathways [36]. Our test demonstrated that high concentrations (100 *μ*g/mL) of C_2_S and TCP particles produced more ROS than the low concentrations (10 *μ*g/mL). This may imply that we should choose lower concentrations of these two biomaterials when used clinically.

RT-qPCR was used to test the TLR2, TNF-*α*, IL-6, and IL-1*β* gene expression levels and ELISAs were used to test the production of TNF-*α*, IL-6, and IL-1*β* proinflammatory cytokine levels. We found that C_2_S particles will exhibit a proinflammatory response by producing TNF-*α* when cocultured with the murine RAW 264.7 macrophage cell line. At the same time, C_2_S significantly increased the TLR2 mRNA expression. When we added siRNA-TLR2-1 to the experimental group, the TLR2 and TNF-*α* mRNA expression levels decreased, which may be a first step to determine that the proinflammatory response mechanism of C_2_S may be related to TLR2-mediated inflammatory pathways. Next, we determined the relative protein expression and found that the p-I*κ*B and p-JNK1 + JNK2 + JNK3 expression levels following 100 *μ*g/mL C_2_S particle exposure increased. Additionally, we also added siRNA-TLR2-1, and the levels remarkably decreased. By chance, we found that the 100 *μ*g/mL TCP particle induced high p-I*κ*B expression levels, which suggests that TCP induces TNF-*α* through the p-I*κ*B pathway. Then, we continued testing the MyD88 levels and found that there were no significant differences. We have evidence to believe that C_2_S induces proinflammatory responses through TLR2-mediated NF-*κ*B and p-JNK1 + JNK2 + JNK3 pathways in the murine RAW 264.7 macrophage cell line; however, TLR2 might not be the only pathway to induce proinflammatory mediators.

Toll-like receptors (TLRs) are mainly expressed in phagocytes and antigen presenting cells, such as neutrophils, macrophages, and dendritic cells (DCs) [37]. TLRs represent a diverse family of molecules that play a critical role in activating the innate immune system in response to pathogens [38, 39]. TLR1 and TLR2 are involved in epithelial homeostasis, and a role for TLR6 in increasing intestinal inflammation in response to pathogen-sensing has been observed [40]. A new class of TLR2 ligands that are produced by *P. gingivalis* likely play a significant role in mediating inflammatory responses both at periodontal sites and, potentially, in other tissues where these lipids might accumulate [41]. In this study, we found that the TLR2 expression was high, and we speculate that C_2_S induces a proinflammatory response. Then, we added siRNA-TLR2-1 to prove it. Moreover, TLR2 plays an important role in the retinal microglial innate response to *S. aureus*, and its sensitization inhibits inflammatory response while enhancing phagocytic activity [42]. The adult cognitive behavior can be influenced, in part, by activation or alterations in the TLR2 pathway at birth [43]. It is thus clear that high TLR2 levels may not only represent a proinflammatory response but may also represent an untoward effect, such as influencing cognitive behaviour.

The nuclear factor *κ*B (NF-*κ*B) is a nuclear transcription factor that regulates the expression of a large number of genes that are critical for the regulation of apoptosis, viral replication, tumourigenesis, inflammation, and various autoimmune diseases. The activation of NF-*κ*B is thought to be part of a stress response as it is activated by a variety of stimuli that include growth factors, cytokines, lymphokines, UV, pharmacological agents, and stress. In its inactive form, NF-*κ*B is sequestered in the cytoplasm, bound by members of the IkB family of inhibitor proteins, which include I*κ*B*α*, I*κ*B*β*, I*κ*B*γ*, and I*κ*B*ε*. The various stimuli that activate NF-*κ*B cause I*κ*B phosphorylation, which is followed by its ubiquitination and subsequent degradation. NF-*κ*B was discovered in 1986 by David Baltimore, who worked in the Cancer Research Center of the Massachusetts Institute of Technology in the US, and Ranjan Sen, who worked in the Whitehead Institute for Biomedical Research [44]. NF-*κ*B plays a critical in regulating function in gene expression by inducing cytokines, and the genes it regulates encode the acute phase response proteins, cytokines, cell adhesion molecules, immunomodulatory molecules, cancer genes, growth factors, and transcription and growth control. By regulating the expression of multiple genes, NF-*κ*B participates in a variety of biological processes, such as immune responses, inflammation, apoptosis, and tumourigenesis.

The C-Jun amino terminal kinase (JNK) family, discovered in 1990, was the observed mitogen-activated protein kinase (MAPK) superfamily member, and it evolutionarily belongs to the conservative serine/threonine protein kinase [45]. The JNK signaling pathway, centered on JNK, can be activated by a variety of factors, such as cytokines, growth factors, and stress (such as ionizing radiation, osmotic pressure, heat shock, and oxidative damage). Many studies suggest that the JNK signaling pathways play an important role in cell differentiation, apoptosis, and stress reactions, and its occurrence and development are involved in a variety of human diseases. Therefore, the JNK signaling pathway is important in regulating the normal and disease status of target cells.

Recent studies found that the MAPK family members (JNK, p38, and ERK) and AP-1 (c-Fos and c-Jun) are well known to be essential to osteoclast formation and proinflammation [46, 47]. The p-JNK may be related to chronic kidney damage and immunotoxicity [48, 49]. On the other hand, NF-*κ*B and JNK are bound up with ROS [50, 51]. These studies implied that C_2_S might increase ROS and TNF-*α* by activating the NF-*κ*B and JNK pathways.

Wear particles induce periprosthetic inflammation and osteolysis through the activation of NF-*κ*B [52]. The suppression of chronic inflammation via the inhibition of NF-*κ*B activity in patients with malfunctioning joint replacements may be an effective strategy to mitigate wear particle-induced periprosthetic osteolysis [53]. Calcium phosphate particles stimulate NF-*κ*B activity [54]. Moreover, titanium particles induce inflammatory osteolysis through the NF-*κ*B and JNK pathways [55, 56]. Like the ideal biological material-titanium particles, C_2_S also induces inflammation. Our study implies that low C_2_S and TCP particle concentrations may be safer than the high concentrations, and it can further reduce common adverse reactions of ROS and NF-*κ*B and JNK pathway activation.

Our study proves that C_2_S may induce proinflammatory responses through TLR2-mediated NF-*κ*B and JNK pathways in the murine RAW 264.7 macrophage cell line; however, the other definite effects of C_2_S in RAW 264.7 cells have not been studied, much less the response of C_2_S in human macrophages. Further studies with C_2_S should be conducted. This study provides a reference for probing the proinflammatory response mechanism of C_2_S. These findings provide some degree of evidence to support the wide use of C_2_S in prosthesis, dental implants, and bone substitutes.

## 5. Conclusion

In this study, we demonstrated that C_2_S particles are not cytotoxic against RAW 264.7 macrophages and that they increase ROS levels. When cocultured with RAW 264.7 cells, C_2_S particles increased the expression of TLR2 and TNF-*α*. Furthermore, C_2_S had no obvious influence on IL-1*β* and IL-6 expressions. C_2_S induced its proinflammatory response through TLR2-mediated NF-*κ*B and JNK pathways in the murine RAW 264.7 macrophage cell line. These results indicate that similar to TCP, C_2_S might be a safe biomaterial; however, it still produces a potential proinflammatory response. siRNA-TLR2 could decrease proinflammatory responses to some degree. Therefore, these findings provide evidence supporting the choice and widespread use of C_2_S as a biomaterial for prostheses and dental implants as well as for use with bone substitutes.

## Figures and Tables

**Table 1:** siRNA target sequences used for quantificational real-time PCR.

Name	Target sequence
siRNA-TLR2-1	GTCCAGCAGAATCAATACA
siRNA-TLR2-2	GCAGGCGACAACCACTTTG
siRNA-TLR2-3	GGAGTCTCTGTCATGTGAT

**Table 2:** Primers used for quantificational real-time PCR.

Name		Primer
GAPDH		AAGAAGGTGGTGAAGCAGG
		GAAGGTGGAAGAGTGGGAGT
TLR2		CTGTGTTCGTGCTTTCTG
		AGGTAGGTCTTGGTGTTCATT
TNF-*α*		TTGTTGCCTCCTCTTTTGCT
		TGGTCACCAAATCAGCGTTA
IL-1*β*		CAGGCAGGCAGTATCACTCA
		TGTCCTCATCCTGGAAGGTC
IL-6		CCGGAGAGGAGACTTCACAG
		TCCACGATTTCCCAGAGAAC
